# Supplementary material for: Insomnia prehabilitation in newly diagnosed breast cancer patients: Protocol for a pilot, multicentre, randomised controlled trial comparing nurse delivered sleep restriction therapy to sleep hygiene education (INVEST trial)
Source: PLoS One. 2024 Aug 14;19(8):e0305304. doi: 10.1371/journal.pone.0305304 (PMC11324102; doi:10.1371/journal.pone.0305304)
Supplement: S2 Table — (DOCX) [file pone.0305304.s002.docx]

Supplementary Table 2. Template for Intervention Description and Replication (TIDieR) checklist

| **Template for Intervention Description and Replication (TIDieR) checklist** | |
| --- | --- |
| **Name of Intervention** | **Sleep Restriction Therapy (SRT) for Acute Insomnia in Newly Diagnosed Breast Cancer patients** |
| Why? | Cancer diagnosis can precipitate the emergence of acute insomnia. We know that cancer patients are at risk of developing chronic insomnia over the course of their illness and beyond. There are several known factors for maintenance of insomnia: irregular bed and wake schedules and spending excessive amounts of time in bed. Prolonged time in bed awake perpetuates the development of insomnia because bed-sleep association decreases over time, in turn generating arousal and fragmented sleep. SRT aims to: 1) restrict time in bed with a view of increasing sleep efficiency; 2) regularise the timing of the sleep-wake cycle; and 3) recondition the bed-sleep association. This trial aims to see if administering SRT as a prehabilitative measure can prevent the development of chronic insomnia. This pilot trial will show whether this intervention is feasible and acceptable to patients during acute cancer treatment phase. |
| What: materials | Materials for patients: patients will receive a SHE booklet, online sleep diary, actigraphy watch and actigraphy watch instructions, a workbook to support at-home implementation of SRT.  Materials for nurses: After a 4-hr training session, nurses will be provided with a folder which contains background information on sleep, insomnia (including its development and maintenance) and SRT. They will also receive a list troubleshooting prompts based on specific common patient scenarios. Nurses will be provided with access to two recorded videos that give an overview of insomnia and SRT implementation. Nurses will be provided with a power-point slide set to work through with each patient during session 1. They will also work through a structured checklist (completed online) for each session to guide content and structure. |
| What: procedures | In session 1, which will be a face-to-face session lasting around 30 minutes, the nurse will work through a Power-Point slide deck with the participant. In this session the nurse will introduce the rationale for SRT, review participant’s baseline sleep diaries, select new bed and rise-times for the coming week based on the baseline diary, offer advice of management of daytime sleepiness (including implications for driving), and discuss any arising barriers/facilitators to implementation.  Sessions 2 and 4 will be brief 10-minute telephone sessions to review progress, trouble-shoot any difficulties and advise upon titration of the sleep schedule.  Session 3 will be another face-to-face session (around 20 minutes) reviewing progress. |
| Who provided: | Research nurses from clinical research networks will be trained to deliver SRT. |
| How provided: | Sessions 1 and 3 are online sessions, sessions 2 and 4 are brief telephone sessions. |
| When and how much | Intervention will be delivered over four sessions. Duration and format of sessions are as follows:  • Session 1 (Zoom/Teams/in person, ~30 minutes)  • Session 2 (by phone, ~10 minutes)  • Session 3 (Zoom/Teams/in-person, ~20 minutes)  • Session 4 (by phone, ~10 minutes)  The treatment will be tailored to each patient’s sleep pattern, following standardised instructions for setting and titrating time in bed (TIB):   \| Criterion \| SRT \| \| --- \| --- \| \| Calculation of prescribed time in bed (TIB). \| Based on average total sleep time (TST) from baseline 7-day sleep diary. Minimum TIB = 5 hrs \| \| Rise time selection \| Time that aligns with working schedule and can be adhered to 7 days a week \| \| Bed time selection \| Typically delayed equalling the prescribed TIB \| \| Weekly adjustments to TIB based on average sleep efficiency for 7 days (SE) (sessions 2- 4) \| a) SE ≥ 85% increase TIB by 15 minutes b) SE = 80-84% no change to TIB c) SE ≤ 79 % decrease TIB by 15 minutes (unless reduction means TIB< 5hrs). Adjustments (advancing or delaying) are typically made to the prescribed bedtime \| \| Napping \| Recommendation to eliminate all napping \|   The nurses will be encouraged to retain a level of flexibility and adapt the TIB prescription is patient is struggling to adhere or cannot tolerate the restriction; patient is excessively sleepy; or a change in health precludes full implementation. In these cases nurses will be encouraged to agree a revised time in bed (increasing in 15-minute blocks) until the patient is content. |
| How well | Sessions 1 and 3 will be audio-recorded and independently appraised for fidelity by an independent CBT-I expert. Nurses follow and complete a checklist during each session to capture various aspects of the sessions, such as content covered, patient’s adherence to treatment instructions, adjusted bed and rise times. |

Supplementary Table 3
