# Supplementary material for: Insomnia prehabilitation in newly diagnosed breast cancer patients: Protocol for a pilot, multicentre, randomised controlled trial comparing nurse delivered sleep restriction therapy to sleep hygiene education (INVEST trial)
Source: PLoS One. 2024 Aug 14;19(8):e0305304. doi: 10.1371/journal.pone.0305304 (PMC11324102; doi:10.1371/journal.pone.0305304)
Supplement: S1 Protocol — (DOCX) [file pone.0305304.s003.docx]

**Study Title:** A pilot randomised controlled trial of sleep restriction therapy versus sleep hygiene education for newly diagnosed breast cancer patients with acute insomnia

**Short Title: INVEST** trial - **IN**vestigating the **V**alue of **E**arly **S**leep **T**herapy

**Chief Investigator:**

Leanne Fleming, PhD

Reader in Psychology

University of Strathclyde

**Funder:** Chief Scientist Office

Version 1.2

4 October 2023

**STUDY TEAM**

**Chief Investigator:**

Leanne Fleming

Reader

School of Psychological Sciences and Health

University of Strathclyde

George Street

Glasgow, G1 1QE

**Co-Investigators:**

Beatrix Elsberger

Consultant Breast Surgeon

Breast Surgery Unit

Aberdeen Royal Infirmary

Forresterhill Road

Aberdeen, AB25 2ZN

James Mansell

Consultant Breast Surgeon

Breast Surgery Unit

Gartnavel General Hospital

Great Western Road

Glasgow, G12 0XH

Simon Kyle

Associate Professor

Nuffield Department of Clinical Neurosciences

University of Oxford

South Parks Road

Oxford, OX1 3QU

David Young

Senior Lecturer

Department of Mathematics and Statistics

University of Strathclyde

Richmond Street

Glasgow, G1 1XH

Kathleen Boyd

Reader

Health Economics & Health Technology Assessment

University of Glasgow

Byers Road

Glasgow, G12 8TB

Vivien Green

PPI Representative

vivienegreentranslations@gmail.com

Contents

[1. Lay Summary 5](#_Toc135829887)

[2. Background 5](#_Toc135829888)

[3. Preparatory Research 7](#_Toc135829889)

[4. Aims 8](#_Toc135829890)

[5. Study Objectives: 8](#_Toc135829891)

[5. Study Design 10](#_Toc135829892)

[6. Participants 10](#_Toc135829893)

[6.1 Inclusion Criteria 10](#_Toc135829894)

[6.2 Exclusion Criteria: 11](#_Toc135829895)

[7. Procedures 11](#_Toc135829896)

[7.1 Patient Screening and Recruitment 11](#_Toc135829897)

[7.2 Randomisation 12](#_Toc135829898)

[7.3 Study Assessment Points 12](#_Toc135829899)

[7.4 Primary and Secondary Outcomes 13](#_Toc135829900)

[7.5 Assessment Tools 13](#_Toc135829901)

[8. Interventions 15](#_Toc135829902)

[8.1 Sleep Restriction Therapy (SRT) 15](#_Toc135829903)

[8.2 Sleep Hygiene Education (SHE) 16](#_Toc135829904)

[9. Qualitative Process Evaluation 16](#_Toc135829905)

[10. Data Analysis 17](#_Toc135829906)

[11. Public and Patient Involvement (PPI): 17](#_Toc135829907)

[12. Adverse Event Ascertainment and Reporting 18](#_Toc135829908)

[12.1 Definitions of Adverse Events, Serious Adverse Events, and Unanticipated Problems 18](#_Toc135829909)

[12.2 Unsolicited/Solicited Assessment and Reporting of Adverse Events and Unanticipated Problems: 18](#_Toc135829910)

[13. Data and Safety Monitoring: 19](#_Toc135829911)

[14. Criteria for Intervention Discontinuation 19](#_Toc135829912)

[15. References 19](#_Toc135829913)

# Lay Summary

Twenty percent of breast cancer survivors have insomnia, which is defined as persistent trouble falling and/or staying asleep that results in difficulty functioning during the day. Sleep difficulties often begin at cancer diagnosis, become worse during cancer treatment, and continue into cancer survivorship. Insomnia interferes with daily activities and may cause other mental and physical health problems. It also makes it more difficult to cope with cancer treatment and makes recovery more challenging. For these reasons, it is important to address early signs of sleep problems in cancer patients by offering interventions that may prevent the development of insomnia. Sleep Restriction Therapy is one such intervention, which helps improve night-time sleep by stabilising sleep patterns and reducing time spent in bed awake. Sleep Restriction Therapy has been used successfully to treat chronic insomnia in breast cancer survivors who have completed cancer treatment. However, it has never been tested on newly diagnosed breast cancer patients with early signs of sleep disturbance, who are undergoing cancer treatment. This study will address this knowledge gap by randomly assigning newly diagnosed patients with poor sleep to receive either Sleep Restriction Therapy or a sleep hygiene education control intervention. We will assess sleep and mental health before and after both interventions to determine how feasible and acceptable Sleep Restriction Therapy is to patients and to plan for a future, larger study. Throughout, we will work with patients with lived experience of breast cancer and poor sleep to ensure our study is informed by their expertise.

# Background

Insomnia disorder (ID) is a causal factor in the evolution and maintenance of physical and mental ill health ([1](#_ENREF_1)), a robust risk factor for all-cause mortality ([2](#_ENREF_2)), and the most commonly reported mental health complaint amongst cancer populations ([3](#_ENREF_3), [4](#_ENREF_4)). Breast cancer patients report the highest rate of ID (~40%) and more circadian disruption than other cancer groups ([3](#_ENREF_3)), which is associated with significantly shorter overall cancer survival, independent of other prognostic factors ([5](#_ENREF_5)). For these reasons, this study aims to explore the feasibility of providing an early sleep intervention to avert the development of ID in breast cancer patients.

Two recent reviews ([5](#_ENREF_5), [6](#_ENREF_6)) synthesise models of insomnia development, the most well-cited of which is Spielman’s diathesis-stress model (1987). This model explains how the stress of cancer diagnosis precipitates the onset of acute insomnia, then altered rest/activity rhythms (i.e., daytime napping, time in-bed extension), implemented to combat the impact of acute insomnia, perpetuate its transition to ID ([7](#_ENREF_7)). Our group have previously completed a programme of work exploring this model of insomnia aetiology in breast cancer ([8](#_ENREF_8), [9](#_ENREF_9), [10](#_ENREF_10), [11](#_ENREF_11)). We reported that 46% of newly diagnosed breast cancer patients develop acute insomnia following diagnosis ([8](#_ENREF_8)). Rates of insomnia remain stable and pervasive for at least 12-months post-diagnosis, indicating that acute insomnia develops a chronic course if not addressed ([8](#_ENREF_8), [11](#_ENREF_11)). We also found that breast cancer patients attempt to compensate for the impact of acute insomnia by engaging in behaviours known to promote the development of ID ([9](#_ENREF_9)). Finally, we reported that ID has a significant negative impact on the psychological and physical wellbeing of people with breast cancer ([10](#_ENREF_10)). Therefore, breast cancer patients would benefit from an early intervention that targets acute sleep disturbance.

Sleep Restriction Therapy (SRT) is an established, single-component treatment for ID and a key ingredient within multicomponent Cognitive Behavioural Therapy for insomnia (CBT-I) ([12](#_ENREF_12)). SRT targets altered rest/activity rhythms, with the aim of increasing homeostatic sleep pressure, over-riding cognitive and physiological arousal, and strengthening circadian control of sleep ([12](#_ENREF_12)). This is because sleep extension leads to a disparity between sleep opportunity and sleep ability, alters the regularity of the sleep-wake schedule, and consequently, increases exposure to light, the chief zeitgeber for the circadian clock. Therefore, whilst SRT may be particularly useful for breast cancer patients with acute insomnia, there are no efficacy studies of SRT in the breast cancer patient population or the acute insomnia population.

One of the strengths of our approach is that it identifies patients with initial sleep disturbance who would benefit from early intervention to prevent insomnia progression. However, robust research is required to evaluate whether SRT is tolerable for newly diagnosed breast cancer patients at such a challenging time. Typically, cancer patients undergoing active treatment are excluded from sleep research studies, so their ability to commit during a time of high psychological impact is unclear. Reassuringly, our Public and Patient Involvement (PPI) group strongly endorse our research plans, and the importance of offering SRT during the acute cancer treatment phase, not least because of the potential for prevention of ID. Finally, assessing intervention acceptability will provide insight into potential optimisation of SRT and recommendations for enhancing future scalability and translation within healthcare. This study will identify and resolve these key areas of uncertainty, informing the design of a larger randomised controlled trial (RCT) and minimise barriers to wider-scale adoption of SRT within cancer care settings.

# Preparatory Research

We have established a sleep and cancer research programme focusing on the aetiology, impact, and non-pharmacological management of insomnia in breast cancer patients. Our research programme has enabled the development of an international breast cancer research register (n=879) and PPI network. Informed by this network, our next step is to evaluate whether we can prevent the development of ID in this at-risk population by offering an early, evidence-based insomnia intervention that targets acute sleep disturbance. Treating acute insomnia has been identified as a priority issue by a task force of experts from the World Sleep Society in their recent guideline for the diagnosis and treatment of insomnia ([13](#_ENREF_13)). Our preparatory pilot work, comprising systematic literature reviews, qualitative studies, observational analyses, RCTs and public engagement work, all validate the proposed study. We recently demonstrated that exhibiting acute sleep disturbance in response to breast cancer diagnosis results in a six-fold increase in insomnia prevalence ([8](#_ENREF_8)) and is a risk factor for the development of ID ([11](#_ENREF_11)). We have also demonstrated that ID is deleterious for psychological and physical wellbeing in breast cancer patients ([10](#_ENREF_10), [14](#_ENREF_14)). Our qualitative studies ([9](#_ENREF_9), [10](#_ENREF_10)) reveal that breast cancer patients implement sleep extension behaviours, intended to compensate for sleep loss, that increase their risk of ID. SRT is an established treatment for ID that works by addressing these sleep extension behaviours to stabilise the sleep-wake schedule. A recent meta-analysis of RCTs by our team demonstrates that SRT significantly improves insomnia severity and sleep continuity in patients with ID ([12](#_ENREF_12)). Therefore, it offers promise as an early therapeutic approach for acute insomnia. Our data also show that insomnia interventions (such as SRT) improve concomitant symptoms like anxiety, depression, and fatigue in breast cancer patients ([14](#_ENREF_14), [15](#_ENREF_15)). In addition, because SRT leads to reduced sleep variability and improved sleep consolidation and quality, it may minimise these other prevalent cancer-related side effects as they emerge, so sleep is a promising transdiagnostic therapeutic target for wellbeing more generally. However, no research has evaluated SRT for acute insomnia in cancer patients, so it is unclear whether it is tolerable and efficacious for patients undergoing cancer treatment.

# Aims

The primary aim of this project is to assess the feasibility and inform the design of a full-scale RCT of SRT for newly diagnosed breast cancer patients with acute insomnia. Secondary aims of this project are to assess the effects of SRT alongside a sleep hygiene education (SHE) control on sleep, rest-activity rhythms, and mental health.

# Study Objectives:

1. To determine rates of participant recruitment and retention
2. To determine intervention fidelity
3. To determine outcome measure completion
4. To determine intervention acceptability

We wish to formally consider issues of recruitment and retention, fidelity, completion of outcome measures, and acceptability, to inform the future development of a definitive multi-centre RCT. Our success criteria for moving forward to full trial are based on a similar pilot RCT of breast cancer patients with insomnia ([16](#_ENREF_16)) and a RCT of Sleep Restriction Therapy in primary care ([17](#_ENREF_17)).

Table 1. Overview of objectives and success criteria

| **Objective** | **Quantitative success criteria** | **Quantitative measurement** | **Timepoint(s) of evaluation** | **Qualitative insights (evaluated throughout)** |
| --- | --- | --- | --- | --- |
| **Recruitment and retention rates** | Recruitment - 80% of target achieved | Enrolment logs for all consenting patients. Pre-screen failure logs for patients who meet inclusion criteria but are not enrolled | Screening | Understanding trial implementation and participation / offer of uptake |
|  | Retention -  <30% attrition rate | Completion of outcome measures | 12 weeks post randomisation |  |
| **Intervention fidelity** | Therapist adherence - >70% of SRT elements covered during sampled sessions | Independent clinician review of recorded SRT sessions and  completion of fidelity rating scale | 6 weeks post randomisation | Understanding therapist and patient perspectives on delivery and receipt of SRT sessions |
|  | Patient engagement -attendance at 2/4 SRT sessions | Intervention attendance logs | During intervention phase |  |
|  | Control group contamination - <10% of SHE patients receive SRT through nurse therapist | Client Service  Receipt Inventory | 6 weeks and 12 weeks post randomisation |  |
| **Outcome measure completion** | >70% of questionnaires fully completed | Completion of outcome measures | Baseline, 6 weeks and 12 weeks post randomisation | Explore the acceptability of the outcome measures for clinicians and patients |
| **Intervention acceptability** |  |  |  | Exploration of cancer journey, service ecology, role of early insomnia intervention and experiences of SRT or SHE. |

# Study Design

A pilot, two-centre, two-arm, parallel group, block randomised (1:1) controlled trial will compare SRT with a SHE control. Both groups will continue to receive standard breast cancer treatment as usual (TAU). Consistent with the requirements of a pragmatic trial, there will be no limitations upon usual care for either group. In this way, the trial represents a comparison of SRT+SHE (+TAU) vs. SHE (+TAU), permitting clear judgements to be made regarding the relative clinical utility of SRT. Due to the nature of the SRT intervention, the therapist and patients cannot be blinded to treatment allocation. However, the statistician will be blinded to treatment allocation (i.e., SRT or SHE) until after analysis is complete.

# Participants

Our target population is newly diagnosed, non-metastatic patients whose primary cancer treatment is surgery. Main study exclusion criteria are limited to conditions contraindicated for SRT or factors that would preclude implementation of SRT. SRT is contraindicated in people with dementia / mild cognitive impairment, epilepsy and psychosis because these conditions can be exacerbated by sleep deprivation, which is a common side effect of SRT. If it becomes clear that a participant has lost capacity during the study, they will be withdrawn from the study but any data they have contributed will be retained and used in the analyses.

- 1. Inclusion Criteria

1. Participant is willing and able to give informed consent
2. Aged 18 years and above
3. Screen positive for acute insomnia, defined as dissatisfaction with sleep quality or duration, accompanied by other night / daytime symptoms, present for between 2 weeks and 3 months
4. Newly diagnosed with non-metastatic breast cancer
5. Primary cancer treatment is surgery
   1. Exclusion Criteria:
6. Pregnancy
7. Additional sleep disorder diagnosis (e.g., restless legs syndrome, obstructive sleep apnoea, narcolepsy) or screen “positive” for additional sleep disorder at study screening interview
8. Dementia / Mild Cognitive Impairment
9. Epilepsy
10. Psychosis (schizophrenia, bipolar disorder)
11. Current suicidal ideation with intent or attempted suicide within past 2 months
12. Night, evening, early morning, or rotating shiftwork
13. Current / previous psychological treatment for insomnia during the last 12 months
14. Chemotherapy and/or radiotherapy commenced

# Procedures

- 1. Patient Screening and Recruitment

Adults will be recruited consecutively from breast cancer results clinics in NHS Greater Glasgow and Clyde and NHS Grampian. Our target population is newly diagnosed, non-metastatic patients whose primary cancer treatment is surgery. Participants can be recruited into the study at any point between cancer diagnosis and the onset of chemo / radiotherapy. Potentially eligible patients will be identified by surgical teams at the breast cancer results consultation. The breast cancer research nurse will inform potentially eligible patients about the study, and if they are interested in participating, they will take the patient through a brief screening questionnaire to confirm initial eligibility (e.g., sleep status, age, not currently pregnant). We have included this brief screening questionnaire prior to signing the consent form based on feedback from our PPI representatives. They felt it was unnecessary (and burdensome) to read the participant information sheet, only to then be excluded by simple criteria that could easily and quickly be assessed early in the study (e.g., current pregnancy). Since patients are not enrolled in the study until they sign the consent form, any data provided until this point will be deleted.

If patients do meet the first set of inclusion criteria, the nurse will pass their contact details on to the research team. The study researcher will then contact the patient to discuss the trial in more detail, offering an opportunity for questions to be asked and answered. If potentially eligible patients express willingness to participate, they will be sent an online participant information sheet and consent form. To ensure we engage with people for whom the study may be both relevant and suitable, the main study exclusion criteria will be very clear in the participant information sheet. If interested in participating, the patient will be required to electronically sign the consent form, marking enrolment into the study, and the second stage of screening will commence.

The second stage of screening involves the completion of a semi-structured interview (on Qualtircs or administered by the researcher over Zoom/Teams or telephone) to establish full study eligibility. This includes assessment of current sleep status (based on diagnostic criteria for acute insomnia outlined in 6.1 above), current psychiatric symptoms and review of other relevant medical information (diagnosis of other sleep disorders, other medical and psychiatric disorders). This is to confirm the diagnosis of acute insomnia and establish that there are no medical or psychiatric conditions that might preclude someone from taking part in the study. During screening, we will also give each participant the opportunity to sign a health release form. This will give us permission to contact their health care provider of choice (e.g., their GP) with information that has been uncovered as part of their participation in this study (and only this information e.g., uncovering potential Major Depressive Disorder), that might warrant further follow-up but that cannot be provided as part of this trial. This will be an opt-in opportunity, and not signing the health release form will not have any impact on their participation in the study. In cases where the participant discloses suicidal ideation or physical/sexual abuse to them or others, the respective authorities will be contacted by the CI even if consent for this action is not provided by the participant. This is made clear to the participant in the participant information sheet and also verbally prior to screening. Once eligibility is ascertained, patients will be randomly assigned to SRT or the SHE control group.

- 1. Randomisation

We aim to randomise 40 patients (n=20 in each trial arm), and conservatively estimate that we will recruit 5 patients per month across both sites in a 10-month recruitment window (assuming a dropout rate of 20%). This represents <10% of the total number of patients seen per month in these clinics. NHS Greater Glasgow and Clyde and NHS Grampian serve a combined population of around 1.7M. In the NHS GGC South Sector breast service, based at Gartnavel General Hospital, there are 550 new breast cancers each year, with over 60% of this population undergoing surgery as their primary treatment. In NHS Grampian, 545 new breast cancers (screening and symptomatic pathways) were diagnosed in 2021. About 84% of these newly diagnosed patients received surgery as their primary treatment. Of these, we conservatively estimate that 40% will meet criteria for acute insomnia (n=315). From these, we aim to randomise patients to take part in the study.

- 1. Study Assessment Points

After randomisation to SRT or SHE, patients will be sent an email with a link to complete a baseline (pre-intervention) assessment of sleep, fatigue, and mental health (questionnaires can be completed over the telephone or on paper should the participant prefer this). Patients will also be required to complete the consensus sleep diary and wear an actigraphy device for 7 days prior to commencement of SRT/SHE to record sleep and rest/activity rhythms. Within-treatment session outcomes for SRT will also be explored using sleep diaries and actigraphy. After completion of the baseline assessment, patients will either be referred to the nurse therapist for commencement of the SRT protocol or provided with the SHE resource. At 6 weeks, and 12 weeks post-randomisation, patients will be requested to complete the same battery of questionnaires as during the baseline assessment. Following completion of the final study assessment (12 weeks post-randomisation), those randomised to the SHE condition will be offered the opportunity to complete the SRT protocol without the requirement to complete any further assessments.

- 1. Primary and Secondary Outcomes

As this is a feasibility study, our primary outcome relates to issues of recruitment and retention, fidelity, completion of outcome measures, and acceptability. Quantitative and qualitative measurement of each of these primary outcomes are detailed in table 1 (section 5) above. Our secondary outcomes relate to improvement in sleep, circadian rhythm, and mental health following SRT. We will evaluate improvements in insomnia severity at 6 weeks and 12 weeks, assessed by the Insomnia Severity Index (ISI), which is valid and sensitive to change in insomnia severity in cancer patients. We will also analyse improvements in mental health [depression (PHQ-9); anxiety (GAD-7)], rest-activity rhythms [fatigue (FACT-F); actigraphy] and estimates of subjective sleep (Sleep Diary). We will also collect EQ-5D-5L data to measure health-related quality of life, from which quality adjusted life years (QALYs) can be calculated.

- 1. Assessment Tools

1. *Insomnia Severity Index (ISI):* The ISI is a brief 7-item scale that measures insomnia severity. It has adequate internal consistency with evidence supporting concurrent, predictive, and content validity.
2. *Patient Health Questionnaire 9 (PHQ-9):* The PHQ-9 is a self-administered screening tool for assessment of the severity of depressive symptoms. The questionnaire has good reliability and validity. It is a simple, brief and reliable tool for the screening and evaluation of depression.
3. *Generalised Anxiety Disorders 7 (GAD-7):* The GAD-7 is a brief and reliable scale that measures the severity of anxiety symptoms. It is appropriate for use with cancer patients and has good reliability and validity.
4. *):* The FACT-F is questionnaire designed to assess fatigue in people with cancer, with good reliability and validity data.
5. *EQ-5D-5L:* This scale is a validated measure of health-related quality of life, from which quality adjusted life years (QUALY) can be obtained. One of the aims of a future definitive RCT will be to include a cost-utility evaluation, therefore we would like to collect some preliminary data now to determine effect sizes for a full-scale RCT.
6. *Consensus Sleep Diary:* Prospective daily diary entries will be used to assess self-reported sleep patterns over a 7-day period. Sleep diaries are a reliable and valid index of insomnia and have been used as an outcome measure in efficacy studies on insomnia. During the baseline, intervention, 6 week and 12 week assessment, prospective self-report data will be collected using the consensus sleep diary. This consists of an assessment of bedtime, sleep onset latency, wake time after sleep onset, rise time, total sleep time, ratings of sleep quality and napping behaviour.
7. *Actigraphy:* The actigraphy device is a wristwatch like device that measures activity through an accelerometer (motion sensor that records the occurrence and degree of motion). Reduced activity can be used as a proxy for sleep, thus providing information about the sleep-wake cycle.
8. *The Client Service Receipt Inventory (CSRI):* The CSRI is a research tool that collects information on service utilisation, income, accommodation and other cost-related variables.
9. *Patient Satisfaction with Treatment:* An adapted version of the Patient Satisfaction Questionnaire Short Form (PSQSF) will be used to assess patient’s treatment satisfaction with the intervention.
10. *Safety:* Adverse events unrelated and related to the interventions will be recorded during treatment. A side-effects inventory, which was developed for individuals with insomnia undergoing behavioural treatments, will be used. Serious adverse events such as deaths, suicide attempts, and serious accidents will also be recorded. We will also encourage patients to contact us with any changes to their health and wellbeing.

Table 2: Overview of scheduled study assessments

| **Measures** | **Screening** | **Baseline (T0)** | **During SRT** | **6 weeks post-randomisation** | **12 weeks post-randomisation** |
| --- | --- | --- | --- | --- | --- |
| ISI, PHQ-9, GAD-7, FACT-F, |  | X |  | X | X |
| Sleep diary |  | 7 days | Daily | 7 days | 7 days |
| Actigraphy |  | 7 days |  | 7 days | 7 days |
| Enrolment logs (recruitment) | X |  |  |  |  |
| Fidelity rating scale (therapist adherence) |  |  |  | X |  |
| Intervention attendance logs (patient engagement) |  |  | X |  |  |
| Client Service Receipt Inventory (control group contamination) |  |  |  | X | X |
| Adverse Events |  |  | X | X | X |
| Treatment Satisfaction |  |  |  | X |  |
| Exit interviews (intervention acceptability) |  |  |  | X |  |

# Interventions

- 1. Sleep Restriction Therapy (SRT)

SRT is a manualised, adaptive, behavioural insomnia intervention that is a key active ingredient within multi-component CBT-I. Our SRT protocol ([18](#_ENREF_18)) involves standardising and (where required) limiting a patient’s time in bed with the aim of increasing homeostatic sleep pressure, over-riding cognitive and physiological arousal, and strengthening circadian control of sleep ([12](#_ENREF_12)). Those randomised to the SRT arm will receive two online sessions supported by two telephone calls over a 4-week intervention phase. Face to face SRT sessions will be available to those who are unable to access online sessions. SRT will be delivered by a nurse therapist, trained by suitably experienced members of our team, during a half-day workshop. Based on patient baseline sleep diary data, the trained nurse therapist will work collaboratively with the patient to encourage good sleep health principles such as not extending time in bed during this stressful period, avoiding napping, and keeping regular bed/rise times etc. For those patients who require it (i.e., those who are already extending time in bed), the nurse therapist will work with the patient to develop a tailored sleep/wale schedule by restricting and adjusting time in bed accordingly.

• Session 1 (30 minutes) – The first session will be an online (Zoom) session. The nurse therapist will introduce the rationale for SRT, review baseline sleep diaries, focus on core SRT principles (not extending time in bed, avoiding napping, keeping regular bed/rise times) and prescribing new bed and rise-times (where required). Advice on the management of daytime sleepiness and discussion of barriers/facilitators to implementation will also be offered. Patients will also be given a booklet supporting home implementation of SRT and SHE guidance [19].

• Session 2 (15 minutes) – The nurse therapist will telephone the patient to check progress, provide an opportunity for questions, and advise on titration of the patient’s sleep schedule for the following week based on a structured algorithm [18].

• Session 3 (20 minutes) - The third session will follow the same structure as session two but will be an online session. The nurse therapist will review patient progress, explore (and attempt to overcome) barriers to implementation, and further titrate the sleep schedule (where required).

• Session 4 (10 minutes) – The nurse therapist will telephone the patient to check progress, provide an opportunity for questions, and advise on further titration of the patient’s sleep schedule if required. Suggestions for ongoing implementation and the management of residual or recurrent insomnia symptoms will also be provided during this final session.

- 1. Sleep Hygiene Education (SHE)

SHE will be delivered via a booklet that provides information about lifestyle changes (e.g., reducing exercise in the evening, light snack before bedtime, reducing caffeine) and changes to the bedroom environment (e.g., dark room, comfortable mattress, optimal room temperature). Patients in the SHE condition will be instructed to implement the SHE advice over a 4-week period. One week after randomisation to SHE, the RA will telephone patients to check they understand the SHE advice and answer any questions they may have. However, the SRT nurse therapist will have no input into delivery of SHE and will not review patients randomised to this condition. SHE has successfully been used as a control condition in other trials evaluating SRT and does not have any therapeutic benefit for individuals with insomnia but is often part of usual care, so is a credible alternative to SRT.

# Qualitative Process Evaluation

To explore both trial and intervention processes, following MRC guidance and using programme theory, we will qualitatively explore contextual factors, implementation processes, and intervention mechanisms of action to fully understand how nurse administered SRT is provided and received in oncology settings. This work complements the quantitative success criteria outlined in Table 1 above. Specifically, we will evaluate how intervention training is delivered to nurses and its acceptability to them, how the intervention is administered by nurses and whether intervention fidelity is maintained, the patient’s response to the intervention, how they interact with it, what they do as a result and any unexpected consequences. We will also explore any contextual factors within clinics and the wider health service environment that affect intervention implementation during the trial, increasing understanding of barriers and facilitators to intervention implementation, providing insights for a future trial and for potential future scale up. To explore these issues, in-depth semi-structured interviews / focus groups (telephone, online, face-to-face) will be conducted by the researcher across both study sites, and we will use theoretical frameworks designed explicitly to understand implementation processes. The sample size for this qualitative process evaluation is 20 [15 patients (10 from SRT arm, 5 from control arm) and 5 clinical staff (including the nurse therapist and those responsible for patient recruitment)].

# Data Analysis

As this is a pilot study, interpretation of the results will be considered with reference to the MRC Framework for Complex Interventions. As they relate to feasibility, no formal sample size calculation has been performed for our primary aim. Details of patient screening, recruitment, retention, withdrawal, adherence, acceptability and follow-up will be reported descriptively as outlined in table 1 above. Success criteria for these domains are summarised as follows: recruitment – 80% of target achieved; retention - <30% attrition; therapist adherence - >70% of SRT elements covered; patient engagement - attendance at 2/4 SRT sessions; control group contamination - <10% of SHE patients receive SRT; outcome measure completion - >70% of questionnaires fully completed.

To fulfil our secondary aim, between group comparisons of ISI 6-weeks post-randomisation will be done using t-tests or Mann-Whitney tests. Based on a mean difference in ISI of 4.2 between groups ([12](#_ENREF_12)) and an estimated standard deviation of 4.5 ([17](#_ENREF_17)) a sample size of 20 in each arm is required to achieve a power of 80% with a 5% significance level (we will aim to recruit 25 patients to each arm of the trial to accommodate our assumed dropout rate of 20%). The results from this feasibility RCT will help to refine the sample size calculation for a future definitive RCT.

# Public and Patient Involvement (PPI):

In preparation for commencement of this trial, we have recruited 5 members of our (previously established) PPI network to help shape the research questions, develop the project design, select suitable outcome measures, advise on the recruitment and data collection strategy, and contribute to proposal writing and editing. This ensures that patient wellbeing and improved patient care are central to our proposed methodology. Two PPI colleagues will be members of the study steering group, and one is a co-applicant on this project. Our PPI network also identified three key groups who will benefit most from the results of this project and will be key targets for our wider collaboration and communication strategy:

1. Patients – our results will inform the development of patient information resources, focusing on prioritising sleep health throughout cancer treatment. This will ensure that newly diagnosed breast cancer patients are informed about ways to minimise sleep disruption whilst undergoing cancer treatment and recovery. It will also open the door for patients to have sleep-focused conversations with clinicians, currently a neglected component of supportive cancer care.
2. Clinicians – our results will enable healthcare professionals who care for breast cancer patients to implement, alter, or augment sleep treatment protocols and signpost patients to relevant support services.
3. Charities – we work in partnership with numerous breast cancer charities, who will publicise our research outcomes on their websites and other patient communication resources. They will use our results to improve staff training programmes, ensuring they are more effectively able to support newly diagnosed patients with sleep difficulties.

# Adverse Event Ascertainment and Reporting

12.1 Definitions of Adverse Events, Serious Adverse Events, and Unanticipated Problems

Adverse events (AEs) will be defined as any unwanted medical event, reaction or side effect that occurs during the research study. Any relationship to study procedures will be determined and each AE defined as either unrelated or possibly, probably, or definitely related to participation in the study. The severity of the AEs will be determined by the type and outcome of the AE. Serious AEs (SAEs) are classified as such if the event results in death, disability, hospitalisation, deformation, congenital abnormality birth defects or any medical events that might lead to the outcomes listed above. The classification of the AE is determined irrespective of the relationship to the study interventions. An adverse event will be classified as an unanticipated problem (UP), if the AE is unexpected, if it is related or possibly related to the study procedures, and if the occurrence indicates that the participants or others are placed at greater risk by completing the study that previously assumed.

12.2 Unsolicited/Solicited Assessment and Reporting of Adverse Events and Unanticipated Problems:

AEs will be assessed systematically using a side effects inventory. Patients will also be encouraged to disclose any AEs, new symptoms or major life events during each assessment (unprompted side effects assessment). Additionally, sleep diaries and self-report questionnaires will be reviewed for any emerging symptoms. All AEs will be recorded in an adverse event reporting form, where a detailed description of the event and follow-up action will be recorded. Additionally, the form will provide an opportunity to record whether the event is classified as serious, whether it is related to the study procedures, and whether it is an unexpected AE (see definitions above). The adverse event form will be passed to the CI for review. All SAEs will be reported to the Ethics Committee within 48hrs and UPs that do not meet criteria for SAEs will be reported to the CI within 48hrs, who is responsible to reporting to the Ethics Committee within 30 days. All remaining AEs will be collated for the steering group committee.

# Data and Safety Monitoring:

An internal steering group committee, consisting of the study team, the patient advisory panel members and one external independent advisor (Professor Paul Flowers, University of Strathclyde) will review subject safety and data collection. The committee will have biannual meetings, where participant safety and data collection will be discussed. This includes AE reporting, withdrawals and dropouts from the study and progress with data collection.

# Criteria for Intervention Discontinuation

This study will be stopped prior to its completion if: (1) the intervention is associated with adverse effects that call into question the safety of the intervention (> 20% of participants develop a SAE related to the study); or (2) any new information becomes available during the trial that necessitates stopping the trial; or (3) other situations occur that might warrant stopping the trial. Given previous research on SRT in breast cancer, it would appear unlikely that these thresholds will be met in this study. Any decisions regarding discontinuation of the treatment would be discussed and made in consultation with NHS ethics and steering group committees.

# References

1. Sivertsen B, Hysing M, Harvey AG, Petrie KJ. The Epidemiology of Insomnia and Sleep Duration Across Mental and Physical Health: The SHoT Study. Frontiers in Psychology. 2021;12.

2. Parthasarathy S, Vasquez MM, Halonen M, Bootzin R, Quan SF, Martinez FD, et al. Persistent insomnia is associated with mortality risk. Am J Med. 2015;128(3):268-75.e2.

3. Harrold EC, Idris AF, Keegan NM, Corrigan L, Teo MY, O’Donnell M, et al. Prevalence of Insomnia in an Oncology Patient Population: An Irish Tertiary Referral Center Experience. Journal of the National Comprehensive Cancer Network. 2020;18(12):1623-30.

4. Sharma N, Hansen CH, O'Connor M, Thekkumpurath P, Walker J, Kleiboer A, et al. Sleep problems in cancer patients: prevalence and association with distress and pain. Psychooncology. 2012;21(9):1003-9.

5. Riemann D, Krone LB, Wulff K, Nissen C. Sleep, insomnia, and depression. Neuropsychopharmacology. 2020;45(1):74-89.

6. Van Someren EJW. Brain mechanisms of insomnia: new perspectives on causes and consequences. Physiol Rev. 2021;101(3):995-1046.

7. Innominato PF, Giacchetti S, Bjarnason GA, Focan C, Garufi C, Coudert B, et al. Prediction of overall survival through circadian rest-activity monitoring during chemotherapy for metastatic colorectal cancer. Int J Cancer. 2012;131(11):2684-92.

8. Fleming L, Randell K, Stewart E, Espie CA, Morrison DS, Lawless C, et al. Insomnia in breast cancer: a prospective observational study. Sleep. 2019;42(3).

9. Reynolds-Cowie P, Fleming L. Living with persistent insomnia after cancer: A qualitative analysis of impact and management. British journal of health psychology. 2021;26(1):33-49.

10. Fleming L, Gillespie S, Espie CA. The development and impact of insomnia on cancer survivors: a qualitative analysis. Psycho-Oncology. 2010;19(9):991-6.

11. Rehman A, Drake CL, Shiramizu V, Fleming L. Sleep reactivity predicts insomnia in patients diagnosed with breast cancer. Journal of Clinical Sleep Medicine. 2022:jcsm. 10170.

12. Maurer LF, Schneider J, Miller CB, Espie CA, Kyle SD. The clinical effects of sleep restriction therapy for insomnia: A meta-analysis of randomised controlled trials. Sleep Med Rev. 2021;58:101493.

13. Morin CM, Inoue Y, Kushida C, Poyares D, Winkelman J. Endorsement of European guideline for the diagnosis and treatment of insomnia by the World Sleep Society. Sleep Med. 2021;81:124-6.

14. Fleming L, Randell K, Harvey CJ, Espie CA. Does cognitive behaviour therapy for insomnia reduce clinical levels of fatigue, anxiety and depression in cancer patients? Psycho-Oncology. 2014;23(6):679-84.

15. Espie CA, Fleming L, Cassidy J, Samuel L, Taylor LM, White CA, et al. Randomized controlled clinical effectiveness trial of cognitive behavior therapy compared with treatment as usual for persistent insomnia in patients with cancer. Journal of Clinical Oncology. 2008;26(28):4651-8.

16. Palesh O, Scheiber C, Kesler S, Janelsins MC, Guido JJ, Heckler C, et al. Feasibility and acceptability of brief behavioral therapy for cancer-related insomnia: effects on insomnia and circadian rhythm during chemotherapy: a phase II randomised multicentre controlled trial. British journal of cancer. 2018;119(3):274-81.

17. Kyle SD, Madigan C, Begum N, Abel L, Armstrong S, Aveyard P, et al. Primary care treatment of insomnia: study protocol for a pragmatic, multicentre, randomised controlled trial comparing nurse-delivered sleep restriction therapy to sleep hygiene (the HABIT trial). BMJ Open. 2020;10(3):e036248.

18. Kyle SD, Aquino MR, Miller CB, Henry AL, Crawford MR, Espie CA, et al. Towards standardisation and improved understanding of sleep restriction therapy for insomnia disorder: A systematic examination of CBT-I trial content. Sleep Med Rev. 2015;23:83-8.
