# Supplementary material for: Insomnia prehabilitation in newly diagnosed breast cancer patients: Protocol for a pilot, multicentre, randomised controlled trial comparing nurse delivered sleep restriction therapy to sleep hygiene education (INVEST trial)
Source: PLoS One. 2024 Aug 14;19(8):e0305304. doi: 10.1371/journal.pone.0305304 (PMC11324102; doi:10.1371/journal.pone.0305304)

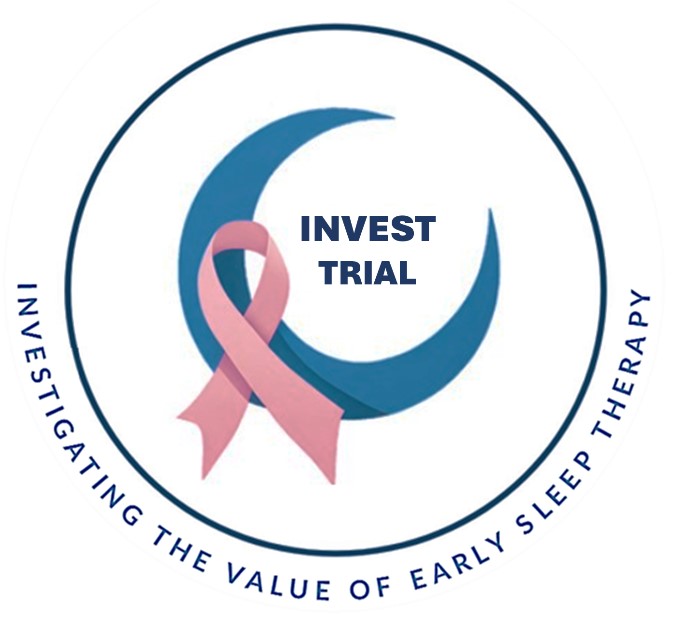


**Patient Information Sheet**

**We would like to invite you to take part in a research trial**

Difficulty sleeping is very common during times of stress and uncertainty. Following a stressful experience, like being diagnosed with cancer, around 50% of people develop trouble falling asleep or staying asleep during the night. For some people, difficulty sleeping only happens occasionally and does not last for very long. However, for others, it can become a more frequently occurring, long lasting problem. This trial compares two different sleep types of non-pharmacological sleep treatments. Before you decide if you want to take part, we would like you to understand why the research is being done and what it will involve for you. Please take the time to read the information in this booklet and talk to others if you wish. If you have any questions, please just ask. Our details can be found at the end of this document.

**Why have I been invited to take part?**

We are inviting people (aged 18 years and over) who have recently been diagnosed with breast cancer, and who are currently having difficulty falling asleep and/or staying asleep, to take part in this trial. **To be eligible for the trial, you should have trouble sleeping at least 3 nights per week, and you should have had this difficulty for no more than 3 months.** We want to compare two different psychological sleep treatments to see whether they help people to sleep better during cancer treatment and beyond. **It is important to note that these treatments are non-pharmacological,** **so you will not be asked to take any drugs or medication as part of your participation in this trial.**

**Do I have to take part?**

No, taking part in the trial is entirely voluntary. If you decide to take part, you will be asked to keep this information sheet for future reference and sign a consent form.

**What will I have to do?**

If you are interested in taking part in this trial, we will first ask you to complete a screening questionnaire (either online, over the phone, or on Zoom/Teams), which will take around 20 minutes. This will help us determine whether the trial is suitable for you. The questionnaire will ask about your sleep as well as your physical and mental health, including a question on suicidality. You will also be given an option to sign a health release form. This form gives us permission to contact a health care provider of your choice (e.g., your GP) about any information you disclose during the trial that might require further follow-up that cannot be provided as part of this trial. However, signing this form is entirely your choice and this decision will not have any impact on your participation in the trial. For you to decide whether you might be suitable for this trial, we have listed the trial exclusion criteria below. If any of these criteria apply to you, then you are **not** suitable for this trial. These criteria are:

- Currently pregnant
- Diagnosis of a sleep disorder other than insomnia (e.g., obstructive sleep apnoea, narcolepsy)
- Diagnosis of dementia, mild cognitive impairment, epilepsy, or psychosis (e.g., schizophrenia, bipolar disorder)
- Night, evening, early morning, or rotating shift work
- Current or previous psychological treatment (e.g., CBT) for insomnia in the last 12 months
- Commenced chemotherapy or radiotherapy
- Feeling that you want to harm yourself

If you decide to take part in this trial and are considered eligible after completing the screening questionnaire, the researcher will notify you by email (or telephone if preferred), which sleep treatment you will receive during this trial.  If it becomes clear during your participation in the trial, that you are no longer able to make decisions for yourself, and/or the trial team have concerns about your ability to provide ongoing consent to the trial protocol, you will be withdrawn from the trial, and any data you have contributed will be retained by the trial team for use in data analyses.

**Sleep treatments**

You will be randomly assigned to one of two sleep treatments: either sleep restriction therapy or sleep hygiene education. This process is like tossing a coin, so you have a 50/50 chance of receiving one or the other treatment. Both treatments offered as part of this trial can be completed alongside any medications you may be taking for your sleep or any other health condition. It is important to be aware that in some people, starting a new sleep treatment can temporarily make you feel sleepier during the daytime. This is a rare side effect that usually settles down within a week or two.

Depending on the sleep treatment you are allocated to, you may be asked to meet with a nurse to discuss your sleep pattern. With your consent, these meetings with the nurse will be recorded to allow us to make sure that the sleep treatment is being delivered as intended. If you are asked to see a nurse, there would be no more than two meetings over the course of a month, and these meetings will be online. However, you can request face to face meetings if you prefer. All other contact will be made over the telephone by either the nurse or the researcher.

**Trial assessments**

Your participation in the trial will last for approximately 4 months. During this time, you will be asked to complete some questionnaires about your health, daytime functioning, and sleep pattern. You will complete the same questionnaires 3 times during the trial regardless of the sleep treatment you receive.  The first questionnaire, called the baseline assessment, is completed shortly after you have been notified of the sleep treatment you will receive. The next two follow-up assessments are completed around 6 weeks and 3 months after the baseline assessment. These assessments will be completed online, using a secure online portal, and should take about 15 minutes each time. If you prefer, the researcher can take you through the questionnaires over the telephone. The researcher will also provide you with an actigraph watch (actiwatch for short), which you will be asked to wear on your wrist for 7 days at around the same time as each of these 3 assessments. An actiwatch measures movement, allowing us to estimate your sleep-wake pattern. The researcher will also ask you to complete a daily sleep diary for the same 7 days that you are wearing the watch. The researcher will give you instructions on how to use the actiwatch and what you need to include in the sleep diary. You will be given a pre-paid envelop to return your actiwatch to the research team. The research team may send you reminders by email, text, or phone to complete and return the questionnaires, actiwatch and sleep diary.

**Optional interview**

You may also have the opportunity to share your experiences of taking part in the trial by giving consent to be interviewed. This is an optional part of the trial, and your decision to be interviewed (or not) does not affect your participation in the other parts of the trial. If you do agree to take part in the interview, a member of the research team will contact you to arrange a suitable time for your interview. Interviews will be conducted by the researcher, either face to face at a convenient location, by telephone, or online (using Teams or Zoom). Each interview will last a maximum of 60 minutes and will be audio recorded. Quotes you provide during the interviews will be anonymised for use in publications from this trial.

**Confidentiality**

All data will be stored securely, according to relevant data protection legislation. Audio recordings and interview transcripts will be stored and transferred securely via password-protected online systems.  All trial information collected will be made anonymous at the earliest practical opportunity. All the information you provide will be coded with a trial identification number so you cannot be identified from it by anyone other than the research team. Responsible members of the University of Strathclyde [and the relevant NHS Trust(s)] may be given access to data for monitoring and/or audit of the trial to ensure that the research is complying with applicable regulations, with your consent. The only exception to this confidentiality clause is if you tell us that you are thinking about harming yourself or someone else. In this situation, the appropriate authorities will be contacted by the research team, even if you have not provided consent for this.

**What will happen to my data?**

We will be using information from you and your medical records to undertake this trial. Research is a task that we perform in the public interest. The University of Strathclyde, as Sponsor, is the data controller. This means that we, as University of Strathclyde researchers, are responsible for looking after your information and using it properly. We will use the minimum personally identifiable information possible. We will keep identifiable information about you for 5 years after the trial has finished. We will store the anonymised research data and any research documents with personal information, such as consent forms, securely at the University of Strathclyde for 5 years after the end of the trial.  Anonymous data will be deposited in an open access repository so other researchers can confirm our results.

The local trial team (based in Glasgow and Aberdeen) will use your name, NHS number, home address, and contact details, to contact you about the research trial, and make sure that relevant information about the trial is recorded for your care, and to oversee the quality of the trial. Your rights to access, change, or move your personal information may be limited, as we need to manage your information in specific ways for the research to be reliable and accurate. You can find out more about how we use your information by contacting the Chief Investigator (details at the end of this document).

**Are there any benefits or risks to taking part?**

You may benefit from improved sleep from taking part in this trial. You will also contribute to research, which may help develop better treatments for people experiencing insomnia following breast cancer diagnosis. There are no known serious side effects from taking part in this trial, however any change to your sleep pattern may be associated with a short-term increase in sleepiness. If you do feel sleepy during the trial, we advise that you avoid activities that require a high degree of vigilance, such as driving or operating heavy machinery.

**What if I don’t want to take part anymore?**

You can leave the trial at any point and the decision to do so will not affect the treatment you receive from your medical team. We would still like to use the data you have already provided, as this will be invaluable to our research. If you have any objection to this, please let us know by contacting us on the details below.

**What if there are any issues?**

For queries about this trial, please contact the trial team on the details below. If you wish to complain about any aspect of the way in which you have been treated during the trial, you should contact the Chief Investigator, Dr Leanne Fleming (contact details below), or the University of Strathclyde Research and Knowledge Exchange office on 0141 548 3707.  The University of Strathclyde, as Sponsor, has appropriate insurance in place in the unlikely event that you suffer any harm as a direct consequence of your participation in this trial. NHS indemnity operates in respect of the treatment with which you are provided.

**What will happen to the results?**

The results of this research trial will be published on the University website, in scientific medical journals and promoted on social media. Your individual results will not be identifiable, nor would you be identified in any report or publication and anonymous data may be shared with other researchers. Should you wish, we will send you a copy of the trial results via your preferred contact method. Anonymous quotes from digitally recorded interviews (optional part of the trial) may be used in publications.

**Who is organising the trial?**

This trial is being funded by the Chief Scientist Office (CSO). It is being conducted by the research team based at the University of Strathclyde, University of Glasgow, and University of Oxford. It is being run across two different regions within Scotland: Greater Glasgow and Clyde and Grampian. All research in the NHS is looked at by an independent group of people, called a Research Ethics Committee, to protect your interests. This trial has been reviewed and given favourable opinion by West of Scotland REC 3 committee (23/WS/0113).

**Contact details**

If you would like to take part in this trial or require any further information, you can contact the research team:

Researcher: Dr Solveiga Zibaite (solveiga.zibaite@strath.ac.uk)

Chief Investigator: Dr Leanne Fleming (l.fleming@strath.ac.uk)

**Thank you for considering taking part**

Once you have considered the information in this form carefully and decided that you wish to participate, please complete the consent form, which is accessible via the QR code or link below:

[**http://tinyurl.com/vhna2mtj**](http://tinyurl.com/vhna2mtj)


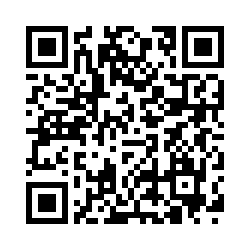

Supplement: S1 File — (DOCX) [file pone.0305304.s004.docx]
